# Editorial: Impact of anesthetics on cancer behavior and outcome

**DOI:** 10.3389/fphar.2022.957070

**Published:** 2022-08-04

**Authors:** Jui-An Lin, Daqing Ma, Szu-Yuan Wu

**Affiliations:** ^1^ Center for Regional Anesthesia and Pain Medicine, Wan Fang Hospital, Taipei Medical University, Taipei, Taiwan; ^2^ Department of Anesthesiology, School of Medicine, College of Medicine, Taipei Medical University, Taipei, Taiwan; ^3^ Pain Research Center, Wan Fang Hospital, Taipei Medical University, Taipei, Taiwan; ^4^ Department of Anesthesiology, Wan Fang Hospital, Taipei Medical University, Taipei, Taiwan; ^5^ Department of Anesthesiology, School of Medicine, National Defense Medical Center, Taipei, Taiwan; ^6^ Department of Anesthesiology, School of Medicine, Chung Shan Medical University, Taichung, Taiwan; ^7^ Department of Anesthesiology, Chung Shan Medical University Hospital, Taichung, Taiwan; ^8^ Department of Surgery and Cancer, Division of Anaesthetics, Pain Medicine and Intensive Care, Faculty of Medicine, Imperial College London, Chelsea & Westminster Hospital, London, United Kingdom; ^9^ Department of Food Nutrition and Health Biotechnology, College of Medical and Health Science, Asia University, Taichung, Taiwan; ^10^ Big Data Center, Lo-Hsu Medical Foundation, Lotung Poh-Ai Hospital, Yilan, Taiwan; ^11^ Division of Radiation Oncology, Lo-Hsu Medical Foundation, Lotung Poh-Ai Hospital, Yilan, Taiwan; ^12^ Department of Healthcare Administration, College of Medical and Health Science, Asia University, Taichung, Taiwan; ^13^ Graduate Institute of Business Administration, Fu Jen Catholic University, Taipei, Taiwan

**Keywords:** local anesthetics, inhalation anesthetics, intravenous anesthetics, neoplasms, behavior, treatment outcome

In two review articles, the mechanisms of cancer inhibition by local anesthetics were reviewed in detail. Zhang et al. summarized the possible pathways in a schematic figure, which provided a framework for researchers to follow when one would like to investigate the impact of local anesthetics on cancer behaviors and associated mechanisms. Generally speaking, local anesthetics involve activating the death signaling and inhibiting survival pathways. According to the current results, their roles seem not independent, more likely to be modulating the chemotherapy during cancer treatment. Furthermore, Zhang et al. pointed out some of the solutions and research priorities, such as standardization of experimental methods, investigation of the stemness of the cancer cells, identification of the specific tumors or types of the cancer cells that may be particularly sensitive to certain local anesthetics, as well as the use of bioinformatics. From the viewpoint of residual cancer cells, Wall and Buggy specifically looked into the effect of lidocaine perioperatively, which has a well-established role either while being infused intravenously or in the provision of epidural anesthesia. Their review provided a state-of-the-art summary of recent advances regarding perioperative lidocaine and its in-depth biological effects from possible aspects. Based on a schematic overview of the pathophysiological mechanisms involved in perioperative metastasis formation, they explained potential tumor-related mechanisms of action of systemic lidocaine during surgery by a model of colonic tumor excision. Although published dosage guidelines may aid in ensuring safe practice, the appropriateness of intravenous lidocaine has recently been questioned in terms of its impact on cancer behavior given as yet inconclusive benefits. Both review articles mentioned that the drug concentrations in most animal and cell experiments were significantly higher than in clinical use. Given the considerable degree of biological heterogeneity between different malignancies, it would be difficult to extrapolate the result of one cancer to another. Different anesthetics promote or inhibit metastasis, depending on the type of tumor cell as well as the type, dose, and regimen of anesthetics used.

This Research Topic also includes some specific analyses, including tumor-specific, anesthetic technique-specific, and anesthetic agent-specific publications to elucidate the association and possibly causal relationship. Most publications on this Research Topic involved female cancers. Liu et al. found that sevoflurane, though with increased migration of 4T1 breast cancer cells, has no difference compared with isoflurane and desflurane on the growth and lung metastasis in the mouse residual tumor model. Chamaraux-Tran et al. provided a prototypical model of combining metabolomics and onco-anesthesia to evaluate the impact of anesthetic on metabolites in triple-negative breast cancer cell lines. Especially for female cancers, ferroptosis might be an emerging target to assess the impact of anesthetics on cancer behavior. Both Zhao et al. and Sun et al. explored anesthetic-specific ferroptosis-related signaling pathways *in vitro* with or without chemotherapeutic agents. In the two retrospective propensity score matching study investigating ovarian cancer surgery Tseng et al. found propofol-based total intravenous anesthesia is associated with better survival compared with desflurane anesthesia, and Zhang et al. reported a positive effect of intravenous infusion of lidocaine on short-term outcomes and survival. However, robust prospective clinical evidence supporting the beneficial anti-cancer effect of intravenous lidocaine treatment is lacking. The scarcity of randomized controlled trials on this topic is also in line with the conclusion made by Luo et al. in this Research Topic. Using the bibliometric method Luo et al. found that the research hot spot in the influence of anesthesia on tumor prognosis mainly focused on retrospective studies over the past 20 years, and the direction is likely in a gradual transition from retrospective studies to prospective randomized controlled trials. Shi et al. conducted a prospective randomized controlled trial comparing propofol-based general anesthesia with local anesthesia in patients with hepatocellular carcinoma undergoing radiofrequency ablation. It would be interesting to know that even propofol-based general anesthesia would aggravate cancer behavior and precipitate pro-inflammatory cytokine secretion from the viewpoint of the patient’s serum. Shi et al. speculated that physiological changes during general anesthesia or accompanying opioid usage during propofol-based anesthesia might be responsible for the results. However, another randomized trial directly evaluating early recurrence by Li et al. showed no difference between paravertebral-propofol-based regional anesthesia and sevoflurane-opioid-based general anesthesia for breast cancer surgery. A trend of reduced recurrence hazard under regional anesthesia in estrogen receptor-negative group, though not significant, warrants an even larger sample to clarify the influence in this high-risk group.

In many *in vitro* studies evaluating the effects of lidocaine or other local anesthetics, the drug was usually incubated with cultured cells for some time, but such a period of stable and direct drug exposure cannot be possible for an *in vivo* experiment when local anesthetics were locally injected unless it is given into the body compartment that would achieve a constant systemic concentration or where local anesthetic can directly expose themselves to the target tumor. Therefore, what we called clinically relevant concentrations would be serum concentration achieved by intravenous (<20 μM) or epidural lidocaine infusion (around 1 μM). Other situations with higher concentrations cannot be called clinically relevant concentrations unless they are administered into specific body cavities, such as intra-bladder or intra-abdominal injection for direct drug exposure. The difficulty in translating the higher concentration results to the clinical setting (such as local injection around the solid tumor) comes from unstable lidocaine exposure to the tumor since the highly soluble lidocaine hydrochloride can be cleared out *in vivo* quickly with rather limited duration. Otherwise, to retain lidocaine at the injection site much longer, lidocaine nanoparticles proposed by Yang et al. can significantly slow down the release rate of lidocaine and could be one of the solutions to help translate the results from higher concentrations studied in the web bench and make it more adequately compatible with the model of local injection around solid tumors. It would be even better if future publications could comment on the effects of lidocaine on cancer behaviors based on clinically relevant concentrations and stratified by body compartments administered, respectively, to help readers evaluate its true impact without being confused by describing the effects according to the results from mixed concentration scales (mM vs μM).

Although all of the above articles described the inhibitory effects of local anesthetics on cancer behaviors, a balanced report cannot be achieved without including the possible aggravating effects of intravenous lidocaine infusion on cancer behaviors. The biphasic effect, especially from drugs, is not uncommon in clinical practice from our previous experience (Lin et al., 2011). It might be premature to deny the possibility that clinically relevant concentrations of lidocaine (<20 μM) could exert opposite effects compared with high concentrations on the millimolar scale. Furthermore, the impact of lidocaine at concentrations corresponding to intravenous (<20 μM) or epidural infusion (around 1 μM) on cancer behavior has rarely been explored. Lidocaine has been proven to consistently promote proliferation in cells derived from metastatic colon cancer (Siekmann et al., 2019). Nevertheless, previous studies have also reported that exposure to 8 μM lidocaine could reduce the barrier property of A549 lung cancer cells using electric cell-substrate impedance sensing (ECIS) technology (Chan et al., 2017). However, the effect of 8 μM lidocaine on cell migration is yet to be clarified. For lidocaine to be infused either intravenously or epidurally, the concentrations to which tumors are directly exposed must be below the toxic concentration (21 μM) to have significance in translational medicine. Contrary to previous reports, our preliminary data showed that lidocaine concentrations corresponding to intravenous infusion in clinical scenarios (1–20 μM) did not affect the growth and proliferation of lung cancer cells but promoted epithelial-mesenchymal transition, which further renders its clinical effects on lung cancer questionable. Including both sides (positive and negative impacts) in the review or discussion section of studies would help achieve a more robust study design in the future.

From describe above and meanwhile to meet the future directions pointed out by Zhang et al., experimental model wise, we call for further studies using clinically relevant concentration (<20 μM) to evaluate the impact of lidocaine on cancer behaviors to mimic stable circulatory lidocaine exposure to the tumors during intravenous or epidural infusion. Cancer stem cell and mechanisms associated with cancer stemness, such as epithelial-mesenchymal transition, would be the future research hotspot in this field. Network analysis using ingenuity pathway analysis corresponding to specific cancer settings might be a practical tool to systematically predict gene alteration according to the wet bench results following lidocaine exposure as a solution to meet the direction toward bioinformatics.
